# Use of next generation sequencing technologies in research and beyond: are participants with mental health disorders fully protected?

**DOI:** 10.1186/1472-6939-13-36

**Published:** 2012-12-20

**Authors:** Iris Jaitovich Groisman, Ghislaine Mathieu, Beatrice Godard

**Affiliations:** 1 Groupe de recherche Omics-Ethics, Programmes de bioéthique, Faculté de médecine, Université de Montréal, C.P. 6128, succ. Centre-ville, Montreal, H3C 3J7, Canada

**Keywords:** Bipolar disorder, Next generation sequencing, Consent form, Return of results, Incidental findings, Genetic counselling, Participants’ protection

## Abstract

**Background:**

Next Generation Sequencing (NGS) is expected to help find the elusive, causative genetic defects associated with Bipolar Disorder (BD). This article identifies the importance of NGS and further analyses the social and ethical implications of this approach when used in research projects studying BD, as well as other psychiatric ailments, with a view to ensuring the protection of research participants.

**Methods:**

We performed a systematic review of studies through PubMed, followed by a manual search through the titles and abstracts of original articles, including the reviews, commentaries and letters published in the last five years and dealing with the ethical and social issues raised by NGS technologies and genomics studies of mental disorders, especially BD. A total of 217 studies contributed to identify the themes discussed herein.

**Results:**

The amount of information generated by NGS renders individuals suffering from BD particularly vulnerable, and increases the need for educational support throughout the consent process, and, subsequently, of genetic counselling, when communicating individual research results and incidental findings to them. Our results highlight the importance and difficulty of respecting participants’ autonomy while avoiding any therapeutic misconception. We also analysed the need for specific regulations on the use and communication of incidental findings, as well as the increasing influence of NGS in health care.

**Conclusions:**

Shared efforts on the part of researchers and their institutions, Research Ethics Boards as well as participants’ representatives are needed to delineate a tailored consent process so as to better protect research participants. However, health care professionals involved in BD care and treatment need to first determine the scientific validity and clinical utility of NGS-generated findings, and thereafter their prevention and treatment significance.

## Background

The use of Next Generation Sequencing (NGS) technologies is expected to greatly facilitate the discovery of many – up till now elusive – causative genes for, and gene variants of, complex trait conditions. Past genetic studies into bipolar disorder (BD) have been characterized by low reproducibility and inconclusive findings [1]. Reasons include a combination of multigenic background and environmental factors, which themselves contribute to a wide range of phenotypic expressions of this condition.

In comparison with traditional sequencing, the use of NGS is regarded as ideal to discover genetic mutations and gene expression variations causative of BD because of the amount and diversity of genetic variants these technologies can reveal [1].

Given the important social and economic burden of BD throughout the world, finding factors that not only help better understand individual predisposition to this condition, but also better prevent the potential harms (such as stigma, damaged self-esteem and anxiety) associated with the risks of erroneous diagnosis and inappropriate therapy will have an enormous social impact. The importance of addressing related ethical issues in concert with efforts to identify such genetic risk factors lies in maximizing health benefits to those affected by this disease while minimizing negative social implications.

The use of NGS in research can provide a great amount of new information, which, at times, is unrelated to the issue that first prompted the study. This has consequences for scientists and their institutions as well as for research participants – inasmuch as there is unexpected information to be dealt with. Researchers are faced with either providing it, or not, and participants with receiving it – or not. BD patients are in a fairly vulnerable position [2] with regard to the significance of results, the difficulty of understanding complex individual findings and, most importantly, because of the impact that learning these results may have on their lives. The latter is heightened in genetic research.

The protection of NGS study participants calls for specific approaches on preserving autonomy, disclosing results and communicating incidental findings [3,4]. However, protocols that address the genetics of mental disorders may well need supplementary social and ethical safeguards for vulnerable populations whose quality of life could greatly benefit from finding underlying causes for their conditions and the development of appropriate treatments.

This article provides a review of the application of NGS to complex traits and its challenges with BD. We discuss the social and ethical implications of the use of NGS in research from the perspective of protecting participants in genetic research protocols directed to subjects with BD. Our approach covers both research and emergent clinical settings requiring special attention.

## Methods

In order to determine the social and ethical issues currently reviewed in the literature related to the identification of gene factors associated with BD, we first performed a systematic review of studies published in the last five years on the ethical, legal and social issues raised by: (1) new genome sequencing approaches, such as NGS, whole exome sequencing, genome-wide association studies, or whole genome sequencing, and (2) genetics and genomics studies on mental disorders, especially on bipolar spectrum disorders. Studies for potential inclusion were identified through a PubMed search, using different combinations of keywords, followed by a manual search. The search was limited to titles and abstracts of original articles, reviews, commentaries and letters. A total of 217 studies were relevant to our project and constituted the sample upon which we performed a detailed analysis of the ethical issues raised by NGS when it is applied to mental disorders, and in particular to BD.

## Results

### NGS in research applied to complex traits

DNA sequencing technologies involving high-throughput, low-cost procedures are expected to greatly help in finding new genomic variations associated with a wide variety of diseases. They are also regarded as the key to comprehending those of multivariate genetic origin [5]. Different alternatives are currently being considered. While the use of Whole Genome Sequencing (WGS) of large patient cohorts is a much-needed approach in complex traits research, so as to detect low frequency genetic variants, it is still being ruled out, due to its high costs [6,7]. Lower-cost possibilities include gene-targeted sequencing, partial sequencing of pooled samples and exome sequencing [6]. The latter is currently the least expensive and a highly reliable alternative to WGS in uncovering genetic defects associated with various complex traits, particularly when combined with sequencing exome flanking non-coding regions [8,9]. Sanger sequencing of these additional regions has also shown their significance [10]. Some genetic variants will be absent or underrepresented using this approach, since approximately 15% of patients with a genetically predisposed condition do not have coding sequence mutations [5]. The application of NGS techniques in research settings appears to be effective, although it displays a number of technical and analytical challenges, ranging from the storing and handling of data or mapping and understanding genetic variations, to differentiating errors and variants of different frequency [6]. Awareness of these issues contributes to the acceptance that complete accuracy is not possible [5,11] and that errors originating in a research context could be reflected in, and transferred to, clinical applications of experimental results. Any consent process for genetic studies using these methodologies and considering communication of research results and incidental findings has to be clear on the limitations of these procedures. It is just as important that health care workers and patients seeking answers about these conditions understand the uncertainties of any possible clinical applications based on results obtained from this technology. We address these issues in detail later in this paper.

### Reasons for diversity

Past genetic studies of complex-trait psychiatric disorders using linkage and association studies have been characterized by low reproducibility and insufficient findings [1]. Two main, recurring aspects of flawed results, i.e., sample size and participant heterogeneity, are still present in large cohort studies conducted with participants diagnosed with BD [12]. While the genotyping of very large samples is expected to be an easily overcome obstacle as NGS becomes more affordable, the heterogeneity of BD subjects remains problematic. BD present a range of subtypes, and the medical diagnostic process itself could be a source of considerable variation [12]. However, the cause of sample heterogeneity, and the reason for the wide range of subtypes in BD and other psychiatric conditions could simply be due to participants’ diverse genetic backgrounds. That is, the same phenotype, or, in other words, the same disease diagnosis, is caused by many genes, or by multiple variants of the same gene(s) [1].

Different and perhaps concurrent scenarios that may help explain BD genetic and phenotypic diversity could be the existence of interactions among genes that are overlooked when genes and gene variants are examined separately; the presence of copy number variants (CNV) that modify how much of the same gene an individual carries – a presence, therefore, that changes the “normal” array of protein-protein and gene-protein interactions; or, still, the variable penetrance of different mutations, in addition to epigenetic changes, which themselves have been causally linked to psychiatric conditions [1,13,14]. This description is indeed common to complex genetic disorders [15].

The application of NGS technology to large scale genetic studies and to small distinctive groups of affected individuals [6] could help identify the many variants that may be simultaneously involved in the different spectrum conditions of BD and in populations of different ethnic backgrounds. Linkage, association and candidate gene approach studies conducted on families, unrelated individuals, and on individuals showing extremes or particular phenotypes of BD have demonstrated that the selection of small distinctive groups of affected individuals could enhance genetic study results [12,16]. Although many of these studies have shown low reproducibility in the past, it is believed the situation would improve if DNA samples of these same cohorts were to be evaluated without predetermined analysis or targeted sequences [5,6,9]. It remains paradoxical that even though patient phenotype heterogeneity remains an issue to be resolved, that very phenotype is at the same time the basis for participant selection when searching for the genetic foundations of BD.

### Disease phenotype

Sample selection is important when seeking to highlight genetic differences that underlie a given condition. Yet it is also influenced by an individual’s desire to participate in genetic research. Some patients with various psychiatric, genetically-based disorders are more inclined than others to seek treatment and to volunteer for clinical studies [17]. While the grounds for this distinctive behaviour are not clear, the behaviour itself may be influenced by a variety of reasons. For instance, identifying genetic ethnic differences between Caucasian and African American Bipolar patients could provide great diagnostic tools for BD [18]. However, fears of racial discrimination, social stigmatization and the breach of privacy associated with mental illness may prompt patients to decide against participation in a genetic study, whatever their ethnic background. Socioeconomic factors such as access to medical, social and financial support or the lack thereof may change a patient’s perception about participating in a research project [19]. Unemployment and health problems are reportedly more frequent with BD than with other psychiatric conditions [20]. Thus, compensation for participation, either monetary or through the greater availability of much-needed treatment, and provided as part of a study’s protocol when otherwise not readily accessible, could be enticing to some individuals, whereas the fear of social stigmatization may deter others from participating in a given research project [2,19]. Taking into account the multifaceted genetic bases of psychiatric disorders, the diverse motivations behind patient participation, or non-participation, in genetic studies, affects the availability of data about the relevant genetic variants which contribute to these disorders, complicating the interpretation of results.

## Discussion

### Consent

Informed consent is one of the most basic elements in the protection of human subjects involved in research projects. While the ultimate goal is facilitating an informed and autonomous decision on participating in research, the intricacy of the protocols and the complexity of the type and later uses of information derived from research require a degree of comprehension that renders many individuals quite vulnerable [2].

Whenever people consent to participate in genetic research, issues such as the scientific bases of the research project, participants’ cultural values and the social position of the targeted study population become particularly relevant [21].

Clear appreciation of the science behind a research protocol could well change the perception of the issue prompting the research study, reducing misunderstandings and potential feelings of stigmatization. In the general population, degrees of comprehension of research issues correlate with levels of literacy and thus with the capacity to better understand the goals, risks and use of study-generated results. That, in turn, is linked to differential health care resources [21]. Lower literacy [21] and lower income – the latter a potential consequence for individuals living with mental health disorders [20] – are in many places associated with restricted access to health care. In such circumstances, access to the genetic counselling necessary to fully understand genetic research results becomes more difficult [21]. Grasping even more complex outcomes, such as those derived from NGS technologies, becomes increasingly problematic.

In terms of the capacity to comprehend the issue that prompts a genetic study and the recourse to NGS, mental health disorders present variable cognitive impairments that can vary over time and between individuals (i.e., “more severe in mania and schizophrenia than personality disorders” [22]). Cognition varies according to a patient’s status at time of consultation. Cognitive impairment has been observed at all stages of BD while decision-making abilities appeared weakened during manic as opposed to euthymic phases [23-25]. Cognition impairment is linked to the effects of medication and the length of the euthymic phase at time of patient evaluation [23,25]. Owen et al. [22] and van der Baan et al. [26] examined the significance of patients’ diminished cognition and how it impacted both treatment decisions [22] and the capacity to give informed consent on sample biobanking for pharmacogenetic studies in psychiatric wards [26]. Decisions on treatment are an imperative in most clinical settings. In many instances, this requirement may possibly be extended to an imminent decision to participate in pharmacogenetic studies when evaluation of drug response relies on genes and gene expression analyses that need baseline sampling or a potential treatment modification decision. The latter may coincide with the need to obtain consent for sample banking procedures [26]. In which case patients experiencing a highly diminished capacity to consent [26] could be asked to understand and decide on the implications of current genetic research technology.

Distinct protective measures should be put in place for any consent process involving NGS technologies and BD, as well as analogous ailments, because of patients’ and participants’ cognitive variability: measures such as educational activities [26,27], the presence of specially-trained personnel [26], and an increased availability of genetic counselling when explaining the purpose of the study and/or the return of genetic results. The extended use of genetic counselling, as reviewed by Bunnik et al. [28], is justified by “The potentially greater psychological impact of genetic testing for psychiatric diseases…” when compared to somatic conditions [28]. We consider that these measures would be decisive in enhancing patients’ and participants’ decision-making capacity.

As discussed by van der Baan et al. [26], appropriate consent forms should be based on opt-in systems so as to safeguard individual autonomy and maintain the integrity of research projects.

### Risks to participants

The consent process considers the impact of research results on the life of participants, on their families and their communities, in particular when the targeted population is an identifiable ethnic minority [21]. These notions have an increased significance in research protocols aimed at discerning genetic predisposition to psychiatric conditions, due to the stigma associated with the latter. Stigma, as discussed by Bunnik et al. [28], may lead to a troubled social life and “discrimination at the work place”.

On consent forms, two risk categories are classified as separate entities, loss of privacy and misuse of information [29], on the one hand, and potential medical and physical harms, on the other. Yet both are interrelated, as study results conveyed with insufficient caution, or the misuse of results through the provision, either of incomplete or wrong information, or one causing a loss of privacy, may themselves cause medical and physical harm by influencing participants’ ultimate behaviour – of particular concern in psychiatric disorders. Receiving results indicating a predisposition to a psychiatric disorder could initiate the expression of first symptoms [28], or could well affect self-image, increase anxiety or induce depression [28,30].

Explanations on confidentiality, participants’ rights of withdrawal and specifications of future use have a different relevance in genetic studies in general, and in the application of NGS, in particular [29]. Data sharing is the basis of current genetic analysis, which limits the possibility of withdrawing, and gives a different perspective to confidentiality, as data and samples may be used under diverse jurisdictions, and future use of data (and samples) becomes complex, because increased knowledge about genetic predisposition shows the intricate relationship between different conditions in addition to generating unsuspected findings. Recent examples are the identification of a previously unknown causative mutation of idiopathic haemolytic anaemia as a result of studying ADHD familial inheritance [31], as well as a common SNP related to comorbid migraine in individuals suffering from BD and ADHD [16].

### Protecting participants

There is a general consensus that the consent process should include an explanation about how information will be handled [3]. How best to ensure protection of the individual and overcome the challenges of respecting confidentiality, the right to withdraw and the proper use of materials and data? It may require the establishment of governance structures involving institutions, Research Ethics Boards and participants’ representatives, with an active input from all parties involved. In addition, a streamlined governance would facilitate tracking the proper use of the great amount of data generated by NGS [29]. We agree with other commentators [4] that input from patients’ representatives and study participants would add the perspective of those individuals most likely to benefit from subsequent research findings. However, we consider that the timing of their participation may vary, as discussed below, in the case of BD, and psychiatric disorders in general. While protective measures as to future use are being established, it is possible to seek a broad consent implying permission for eventual applications, an option already in use in oncology settings [32]. Two other options, keeping genetic research closer to the subject of the original consent, or obtaining a new, updated consent from research participants [33], are also legitimate.

### Feedback, return of results and incidental findings

The question of how to determine what feedback [5] BD research participants should receive about their genomes brings to light the importance of providing measures to protect research participants, their families and communities, considering “the societal and psychological sensitivities that surround psychiatric diseases” [28].

Results can vary in their levels of direct relevance to the issue that prompted the research project at the outset, while incidental findings are generated faster than, in many cases, they can be understood. Return of individual results have been part of the consent process in various oncology settings for some time [32]. It is clear that some areas of research are more closely linked to a therapeutic component than others, making return of individual results easier and more significant. Age of onset, available therapies and preventive treatments for a given health condition should be used to evaluate the ethical implications of returning research results, incidental or otherwise, in particular when mental health is part of the equation. For instance, using these parameters, Bunnik et al. [28] clearly differentiate the psychological and social risks of using genetic test results to convey predisposition to diabetes, age-related macular degeneration and clinical depression. Their results highlight the negative impact on disease progression when these three factors are not clearly defined at the time of sharing results [28].

As for individual wishes and personal motivations for participating in research, another study found that parents of autistic children showed a strong desire to receive research results, independently of their nature, meaning, or relation to the reason that first prompted the study [34]. Although their understanding of the difference between research and clinical settings was clearly stated, their expressed wish for feedback was associated with feelings of “relief, understanding and preparation for the future” [34]. It thus becomes apparent that those who participate in research protocols seek in some way to better understand their condition. Return of individual results may thus distort the aim of the research by generating therapeutic misconception [32].

In Misra et al., BD patients were questioned on their role as research participants. They misunderstood the notions of therapeutic care and research [2], illustrating that participants in BD research protocols can be more vulnerable to therapeutic misconception and can lack autonomous decision-making power. In other words, offering to return research results – independently of their nature – could further heighten the confusion surrounding the difference between research and clinical practice in this population if not accompanied by adequate education and counselling.

A way of respecting participants’ autonomy would be to disclose results in a tailored fashion, following options that Research Ethics Boards (REBs), together with participants and researchers, could choose, according to the subject matter and the social group targeted by the study [4,21]. It has been argued that the promise of returning individual research results will increase the work load for both researchers and the research infrastructure [4]. This becomes particularly true when including the costs of associated genetic counselling. Therefore, recommendations, such as those set forth in the Tri-Council Policy Statement (TCPS) [35], to employ genetic counselling to communicate genetic research findings, may ultimately lead researchers to restrict the communication of individual research findings derived from the use of NGS.

The return of individual results is a subject of debate in many fields of research. In Canada, the TCPS promotes the communication of all material, incidental findings that have “significant welfare implications for the participant, whether health-related, psychological or social” [35]. No such recommendation is made when the study tools are designed for research and have no bearing on clinical diagnosis [29]. The NGS technologies currently used and developed in various research settings do not respond to standards and validation processes required for and followed by procedures in clinical practice [29]. Bredenoord et al. explained that although these positions are contradictory, opposing standpoints regarding the disclosure of incidental findings nonetheless keep the basic human principal of saving a life paramount [3].

There is, however, a basic ethical principle that should prevail, independently of the governance that guides the process of returning results and incidental findings: an evaluation of the risk-benefit ratio for the individual receiving the information. While understanding the benefits seems a simple process, the extent of the risks of communicating incidental findings to individuals and families affected by mental health disorders, risks such as uninsurability [36], stigmatization [28], unpredictable discrimination, and loss of social support, are not always evident. Even when participants are willing to be informed individually, regardless of the nature of the research results [34], the consent process would have to be clear enough to explain to what extent being informed of some outcomes could jeopardize their future.

There appears to be a clear necessity for governance, proper policies and procedures [33], based on the needs and characteristics of different social groups, to manage whether or what information is conveyed to them [3,4,29]; to whom it should be communicated, i.e., to individuals alone or also to families and physicians [29,37]; who is responsible for returning results, solely the investigator conducting the original research or equally those using the data and samples as secondary research material [29]? The direct involvement of institutions and their Research Ethics Boards could be crucial in handling this process [29,33].

Finally, the return of research results is deemed important from a societal standpoint, inasmuch as it contributes to building trust toward research endeavours [29]. However, it is imperative to differentiate results that are public from those conveyed to individuals, whether they be incidental results or ones prompted by the object of research. With an increasing awareness of consumer genetic testing, individuals may gain a different perception of genetic “research” and thus, their views as study participants could possibly change [29]. Previous reports indicate that individuals with mental disorders and their immediate relatives are more prone to undergo genetic testing to determine their disease susceptibility [30,38]. This tendency, in addition to the cognitive impairment associated with mental health conditions, could help explain the possible confusion in reasons given by individuals to become research participants, and strengthens the need for additional educational efforts at time of recruitment. The expectations of those who take part in research protocols with the sole purpose of helping others have to be distinguished from those that hope to obtain genetic information about themselves [29]. The research community is indebted to the altruistic group, while the second one should be considered as a clientele of genetic products and reminded of the exact goals of any research project.

### Informed decision-making and autonomy

There is a very close link between the return of research results and participant autonomy. As discussed above, individuals are selected based on a phenotype for which a genetic cause is not known or because of the intuition that an underlying genetic factor may explain their phenotype. It is on these bases that individuals are regularly invited to participate as subjects in research projects, and, if they accept, asked if they want to know some, or all of the findings, and if they allow the latter to be shared. While P. Bielby explained that the consent process must not have an external form of control, so as to avoid any possible coercion [39], Misra et al. showed that “… over half of all subjects believed that their primary mental health provider could convince them to participate even if they did not want to” [2].

In genetic studies it is possible that a gene associated with a disease phenotype may be discovered while its function is not yet understood, and thus there are no prevention or treatment possibilities [11]. The application of NGS would lead not only to the discovery of causative gene-diseases but also to the identification of countless mutations unrelated to the original goal of the study, and that could yet have health implications for the individual participants, their immediate family and their offspring [11,29]. As previously discussed [39], competence to consent to participate in research implies “that an individual receive sufficient information in order to make that decision in an appropriate way”. However, it is in a context of vague knowledge that researchers ask participants to decide “autonomously” on what it is they want to know, or not know.

Different solutions have been proposed to help respect the basic principles of decision-making and autonomy. One possibility consists of giving individuals the choice of knowing only part of the results, for example by receiving information solely on gene mutations leading to preventable or treatable disorders at the time of discovery, whether related or not to the subject that prompted the study [4]. In the case of minors, information about those conditions medically relevant to their particular age is a valid alternative. The option of receiving different information at successive life stages is also legitimate, although it requires special procedures and resources to store and retrieve data [11], all the while safeguarding participants’ confidentiality and, thus, increasing researchers’ responsibilities [4]. The option of not providing any tests results at all [11] does not comply with regulations that seek to promote the communication of all “material incidental significant findings”, such as the TCPS [35]. This dichotomy has practical solutions at the level of the REBs, when implementing different “consent packages” or considering alternatives for the return of results [4,33].

### Clinically relevant ethical challenges

While the influence of NGS increases, there is a need to examine how it may modify health care practices and what new clinical and ethical challenges may arise.

There are expected benefits associated with the use of NGS technologies in clinical settings for BD, by allowing, for instance, early intervention and, thus, presenting – as described by Leopold et al. – the prospect of reducing the symptomatology and diminishing the negative life consequences that are attached to this condition [20]. NGS could increase the capacity to quickly scan a genome in the search for potential genes, mutations or variations, allowing for better stratification and validation, thus leading to better defined BD subgroups. More reliable and earlier diagnostic tools could then be developed, permitting the development of new and individualized therapies, optimizing treatment responses. However, there are potential socio-ethical risks associated with these expected clinical benefits, such as risks of biohype, therapeutic misconception, genetic exceptionalism vs. denial of the genetic component by individuals who do not have the coding sequence mutations, a lack of genetic counselling resources, a lack of education among health professionals, a lack of social support, inequity of access to health care services, or, still, stigmatization, without forgetting that society’s willingness to pay high costs for drugs is becoming increasingly doubtful.

Initial symptoms of BD may appear as early as mid-adolescence, with a higher prevalence of mood and non-mood disorders in offspring of BD parents as compared to a control population [40]. Tools to define very early signs of the disease would help distinguish different BD subtypes and select proper treatment [20]. We agree with previous reports [20] that the improvement of adolescents’ and young adults’ quality of life, by facilitating completion of studies and strengthening family and social ties, will decrease stigma and increase the social support needed to cope with BD. Based on age of onset and the need to determine early signs of BD, we infer that NGS will impact child, adolescent and adult clinical settings, the latter probably because of the quest for more personalized therapies and the desire to know the genetic susceptibility of their offspring. The influence of NGS on BD in the clinical field is reinforcing a growing need for genetic counselling. Counselling is required to guide and help individuals and families seeking to determine the causative genetic bases of their own or a loved one’s disease, and to apply the proper treatment. It is offered in order to “avoid maleficence and support autonomy” [11], and, as such, it reduces the psychological burden of disclosing untreatable disorders [11]. For instance, when Hereditary Nonpolyposis Colorectal Cancer (HNPCC) is suspected, the course of action involves an extensive process of genetic counselling, and predictive testing is accepted because an effective course of action is available [41]. Uncertainties about medical decisions appear when individuals with a family history of HNPCC do not show mutations in high-penetrance genes [41]. The latter situation resembles the circumstances that increasingly surround countless medical conditions with an already “known” genetic background and it intensifies when – as in many psychiatric disorders – there is no precise knowledge of the underlying causes of the disease, either genetic or functional.

Two major ethical concerns arise from the use of NGS for BD in adolescents, as parents and their children are concurrently involved in making decisions about the right to know or not to know for each. In this context respect for children’s own rights needs further attention. As for using NGS for BD in adults seeking to gain knowledge about how their genetic makeup may influence their descendants, the effect of gene-environment interaction, which could be either positive or negative, and in itself linked to psychiatric conditions, may need careful consideration to avoid further discrimination and inappropriate medical conduct. Genetic counselling will require the allocation of both time and sufficiently trained personnel [11]. To mitigate these needs, the recourse to group counselling and the use of decision support services are thought of as reliable substitutes [11].

Lastly, NGS is already being used in pediatric settings for autosomal and X-linked recessive conditions when symptoms are suggestive but a clear diagnosis is not possible [42]. The application of NGS was accepted as constituting “compassionate use” [42] in a clinical setting for a child of 15 months, and genetic results were corroborated by sequencing in a licensed clinical laboratory [43], as opposed to those obtained through research methodologies that do not follow standardized procedures. In this example, genetic results were corroborated by the functional assays followed in research settings [43], because there was a degree of understanding of the basic role played by the “new gene” involved. These examples demonstrate both the need for standardized procedures to validate results and the need to understand the biological significance of the findings in order to corroborate the outcomes. It also becomes clear that collaboration between institutions and their ethics boards helps support physicians and researchers, while protecting patients who are research participants.

Concurrently, the use of these technologies gives rise to a sequence of ethical and social dilemmas, regarding both the implications of immediate treatment decisions and the extended meaning of incidental findings and deterministic life consequences that could ensue [5]. It is reasonable to think that these difficulties will only be countered with proper regulations – many of them common to the generalized use of NGS in clinical settings – with their emphasis on guaranteeing the rights of all parties involved [11,44]. Delineating the clinical applications of new genetic technologies may require taking into consideration the opinions and beliefs of patients and their families, physicians, ethicists and policymakers [42]. However, the uncertainties are so great, particularly when dealing with diseases having a multivariate component and carrying a strong social stigma, as is the case with BD, that we believe the process of developing such regulations may require that researchers and health professionals consider scientific validation, clinical utility and prevention or treatment first, whether with the help of NGS technologies or not, before including the opinions of affected patients. Interestingly, when patients, physicians and the general public were questioned about the development of pre-symptomatic and prenatal tests in order to determine attitudes towards the availability of genetic tests for BD susceptibility genes, patients and members of the public were in favour of the development of pre-symptomatic – but not prenatal – testing. Psychiatrists, who would administer such tests, appear significantly more cautious, even towards the use of pre-symptomatic genetic testing [38].

## Conclusions

We reviewed some major issues surrounding the protection of NGS research participants, in particular for BD, yet which are also relevant to other psychiatric conditions. Our analysis included general elements of the consent process, such as comprehension of the research per se and its associated risks, risks both inherent to the protocol and derived from omissions in participant protection safeguards. We also discussed, separately, yet in the framework of the consent process, the return of individual results and incidental findings. Finally, we explored the issue of autonomy. These three subjects could and should be further developed, as is the case with topics such as the safeguarding of privacy, highly relevant in its own right, due to the amount of information generated through NGS, and the use of samples and data in subsequent, cooperative work.

The topics of our discussion are among the most important ones for participant protection in the field of genetic research on mental health, and, as we saw with the example of BD, highly relevant because of a direct correlation between participant autonomy, the uncertainties surrounding the causes of such mental health conditions and the cognitive capacity of the targeted population. There is a need to regulate the conduct of research and the subsequent use of data generated by NGS technologies [3,33]. Regulations should be developed through the common efforts of researchers, their institutions, and REBs, on the one hand, and participants’ representatives, on the other, so as to arrive at a tailored consent process able to protect those research participants who suffer from, or risk suffering from, mental health conditions. Active measures, such as educational activities [26,27], the presence of specially trained personnel [26], and an increased availability of genetic counselling can play a decisive role in reinforcing the autonomy of research participants.

We also examined the role of genetic counselling when communicating research results and incidental findings to participants [35], seeing as this process becomes more demanding with NGS research projects [3,33]. Applied to genetic psychiatric research, the counselling process has to account for the limitations of results obtained through NGS technologies, due to the techniques involved, but also to the multivariate origin of psychiatric conditions. The counsellor is also faced with participants’ mixed perceptions and various expectations of research results.

There are emergent clinical applications of NGS. In the case of BD, and with regard to age of onset, there exists a need to better determine early warning signs and improve treatment. We believe this will affect child, adolescent and adult clinical settings, with an even greater emphasis on the need for genetic counselling [11].

While the input of individuals that are directly affected by a given condition is indispensable, in the case of BD, as with mental illness in general, researchers need to determine the scientific validity of findings while the professionals involved in care and treatment need to determine both the clinical utility of those results and their significance for prevention and treatment, and these priorities should be combined when both groups collaborate in preparing guidelines and regulations on using all the data generated by NGS, since this information will exert an ongoing influence on the way clinical practice is carried out.

## Competing interests

GM and BG declare that they have no competing interests. IJG serves as a member and consultant on ethics committees in the public and private sectors.

## Authors’ contributions

IJG carried out bibliography selection and content analysis, manuscript conception and preparation. GM participated in selecting and analyzing bibliography, contributed to draft the manuscript and its design. BG is the corresponding author, conceived of the study and participated in its design and coordination and preparation of the manuscript. All authors read and approved the final manuscript.

## Pre-publication history

The pre-publication history for this paper can be accessed here:

http://www.biomedcentral.com/1472-6939/13/36/prepub
